# Addressing the need for an appropriate skilled delivery care workforce in Burundi to support Maternal and Newborn Health Service Delivery Redesign (MNH-Redesign): a sequential study protocol

**DOI:** 10.12688/wellcomeopenres.17937.2

**Published:** 2022-09-26

**Authors:** Desire Habonimana, Attakrit Leckcivilize, Catia Nicodemo, Mike English

**Affiliations:** 1 Centre de Recherche Universitaire en Santé (CURSA), Department of Community Medicine, Faculty of Medicine, University of Burundi, Bujumbura, 5190, Burundi; 2 Centre for Tropical Medicine and Global Health, Nuffield Department of Medicine, University of Oxford, Oxford, UK; 3 Centre for Health Service Economics and Organisation, Department of Economics, University of Oxford, Oxford, UK

**Keywords:** EmONC, skilled birth personnel, health policy, Burundi

## Abstract

Background

Despite Burundi having formed a network of 112 health facilities that provide emergency obstetric and neonatal care (EmONC), the country continues to struggle with high rates of maternal and newborn deaths. There is a dearth of empirical evidence on the capacity and performance of EmONC health facilities and on the real needs to inform proper planning and policy. Our study aims to generate evidence on the capacity and performance of EmONC health facilities in Burundi and examine how the country might develop an appropriate skilled delivery care workforce to improve maternal and newborn survival.

Methods

We will use a sequential design where each study phase serially inputs into the subsequent phase. Three main study phases will be carried out: i) an initial policy document review to explore global norms and local policy intentions for EmONC staffing and ii) a cross-sectional survey of all EmONC health facilities to determine what percent of facilities are functional including geographic and population coverage gaps, identify staffing gaps assessed against norms, and identify other needs for health facility strengthening. Finally, we will conduct surveys in selected schools and ministries to examine training and staffing costs to inform staffing options that might best promote service delivery with adequate budget impacts to increase efficiency. Throughout the study, we will engage stakeholders to provide input into what are reasonable staffing norms as well as feasible staffing alternatives within Burundi’s budget capacity. Analytical models will be used to develop staffing proposals over a realistic policy timeline.

Conclusion

Evidence-based health planning improves cost-effectiveness and reduces wastage within scarce and resource-constrained contexts. This study will be the first large-scale research in Burundi that builds on stakeholder support to generate evidence on the capacity of designated EmONC health facilities including human resources diagnosis and develop staffing skill-mix tradeoffs for policy discussion.

## Introduction

Burundi aspires to deliver an ambitious maternal and newborn health agenda ^1, 2^ by reducing maternal mortality from 568 deaths per 100,000 live births in 2015 – which ranked among the top fifteen highest maternal mortality ratios in the world ^3, 4^ – to below 140 deaths per 100,000 live births by 2030 ^5^. At the same time, the country aims to halve neonatal mortality from approximately 23 deaths in 2016 ^6^ to below 12 deaths per 1,000 live births ^5^. The major direct causes of maternal deaths include obstetric haemorrhage, eclampsia, and uterine rupture ^7– 9^. Global evidence corroborates with specific local evidence in Burundi. For instance, findings from a recent small-scale study conducted on 184 maternal deaths that occurred in rural hospitals revealed that obstetric haemorrhage was responsible for 72.2% of all-cause maternal deaths and eclampsia and uterine rupture represented 10.3 and 8.2% of maternal deaths; respectively ^10^. To achieve its aspirations, Burundian policymakers will need to strategically allocate resources, redesigning maternal and neonatal health care provision to ensure that all mothers and babies receive adequate intrapartum and postpartum care including emergency obstetric and neonatal care (EmONC). Delivering such services is critically dependent on having sufficient, appropriately skilled human resources who are competent to offer adequate, timely, and life-saving maternal and newborn care ^11– 13^. Today, a network of 112 health facilities provide EmONC services in Burundi. Of them, 59 primary care facilities should dispense basic emergency services and 53 hospitals should provide comprehensive care ^14^. In this country, delivery care is officially only provided by qualified nurses, midwives, medical doctors, or obstetricians depending on the availability of human resources and the need for higher or specialised competency. Except for obstetricians, all these professionals must complete pre-service or in-service EmONC training to qualify as “skilled delivery personnel”. For clarity, we use “maternal and newborn health professionals or cadres” to refer to all qualified and licenced nurses, midwives, medical doctors, and obstetricians and “skilled delivery care personnel or cadres” to imply qualified obstetricians or nurses, midwives, and medical doctors who have completed the EmONC training.

Generally, Burundi has made significant progress in terms of maternal and child health. From 2012 to 2017 for instance, 84% deliveries occurred in a health facility with some areas achieving more than 90% of health facility based deliveries. Many deliveries (77%) were assisted by nurses while only 8% of deliveries were assisted by qualified medical doctors. Despite a persistent high rate of maternal and newborn complications (15% of childbirths) ^15^, it remains unclear whether nurse birth assistants are skilled birth attendants as per the standard definition ^16^. Existing data from the health information system (HIS) in Burundi do suggest a declining trend in maternal and neonatal mortality partly as a result of EmONC interventions. More specific local research also underscores the critical role of EmONC services. A study conducted in a rural district hospital of Burundi demonstrated that 60% of 6,084 mothers needing emergency care underwent a major (42%) or minor (22%) lifesaving surgical procedure ^17^ that in many cases were likely to have averted a death; for example surgery for uterine rupture and extra-uterine pregnancies ^17^. However, a key concern now is whether quality EmONC services are available and accessible to all women including those in rural settings. For effective service provision, sufficient numbers of skilled health workers who have received the necessary training and support to maintain their skillset to national and international standards are needed. National health policies and planning that guide resource allocation should therefore ensure appropriate staffing that takes account of local service demand, needs, and workload.

With attention to human resources for health (HRH), Burundi’s aspirations to deliver on a universal health coverage agenda are undermined by severe shortage and maldistribution of health workers ^18^. With attention to the quantity of trained health personnel for instance, the number of medical doctors per 10 000 population which rose from 0.28 in 2010 to 1.00 in 2017 ^19^ implies that Burundi remains below the recommended World Health Organization threshold of 2.3. skilled health workers (physicians and nurses/ midwives) per 1000 population ^20^. Moreover, for an estimated 11.5 million population, the country has less than 200 medical specialists across all disciplines of whom more than 95% are located in Bujumbura capital city which is home to only 2.7% of Burundi’s population ^19^. Under those circumstances, Burundians continue to face difficulties to access and use quality health care services ^21– 24^. This places Burundi among countries that continue to register higher maternal and neonatal mortality ratios as the reduction of maternal and neonatal deaths requires equitable access to quality health care services which constitute a cornerstone of the survive, thrive, and transform agenda ^1, 25^. Noting that Burundi’s population is mostly female and youthful ^26^, the country needs to decide on how many facilities should offer basic and comprehensive EmONC as well as their geographic distribution and population coverage to ensure equity in quality delivery care access.

Our research stems from the above background and seeks to address some of the above raised concerns. In Burundi, there is sparse empirical evidence on the capacity and performance of EmONC health facilities and the real needs to inform proper planning and policy. Particularly, partial evidence has highlighted that Burundi faces major barriers to quality EmONC service delivery. Some of the challenges that undermine the sustained provision of quality EmONC services include the inefficient resource allocation, shortages, and maldistribution of skilled birth personnel, increasing workloads, and the lack of essential supplies and medications, among others ^27^. A weak training curriculum, a poor harmonisation and coordination of training, and the lack or inadequate in-service training have also been cited ^27^. Therefore, our study aims to generate empirical evidence on the capacity and performance of EmONC health facilities in Burundi and further examine how the country might develop an appropriate skilled delivery care workforce to improve maternal and newborn survival.

### Study aim and objectives

This study aims to explore how EmONC services are currently organised in Burundi, diagnose EmONC human resources issues by focusing on skilled delivery care personnel, and examine how the country might develop an appropriate skilled delivery care workforce to improve maternal and newborn survival. We focus on human resources as this constitutes a major stake in health care service provision. Also, it takes a long time to produce the right workforce if an expansion in service provision is needed, hence the need to explore the most efficient and effective mix of staffing. Specifically, the study will (i) examine the prevailing capacity of designated EmONC health facilities including the scope of emergency care currently being provided, (ii) describe the national total stock of maternal and newborn care workforce and estimate the government budget impact of training and employing different skilled delivery care cadres, and (iii) estimate and cost the workforce gap of skilled delivery care and develop skill-mix staffing alternatives to close the identified gap. Additionally, the study will advise on the need to empower all or some designated EmONC health facilities and whether the country might consider increasing the number and geographic and population coverage to ensure equity. 

## Methods

### Study design

This study will use a sequential design with each of the phased research stages serially inputting into the subsequent phase. Three main study phases will be carried out: i) an initial policy document review to explore global norms and local policy intentions for BEmONC and CEmONC in Burundi with a particular focus on stated or reasonable staffing norms for different levels of facility and ii) a cross-sectional survey of all BEmONC and CEmONC health facilities to map out and determine what percent of facilities are functional including geographic and population coverage gaps, to identify staffing gaps assessed against norms or expectations, and to identify other needs for health facility strengthening. Finally, we will examine potential staffing costs and explore alternative staffing options (service designs) that might best promote service delivery with adequate budget impacts to increase efficiency. Throughout the study, we will engage with a stakeholder group to provide input into what are reasonable staffing norms as well as feasible staffing alternatives within Burundi’s budget capacity.

### Study setting and participants

This study will be carried out in all designated EmONC health facilities in Burundi (n = 112) which comprise 59 primary health facilities providing basic emergency care and 53 hospitals dispensing comprehensive emergency care. We will engage stakeholders including policymakers, researchers, donors, and implementers and target health facility managers, heads of maternity and labour wards or units, skilled delivery care professionals, and students as detailed in Table 1.

**Table 1.** Description of study participants and sample size.

Phase	Activity description	Study participants	Sample size estimation
One	Policy document review to gather evidence on EmONC staffing standards for LMICs	—	—
	Stakeholder panel to reflect on global practice and recommendations and discuss acceptable EmONC staffing standards for Burundi	Policymakers, donors, researchers, and implementers	6–8 participants (Supplement 1). List of stakeholders for the MNH-Redesign project). The stakeholder group was formed by the MoH in consultation with partners namely WHO and JICA and professional bodies namely AGOB (Burundian Association of Gynaecology and Obstetrics) and ABUNE (Burundian Association of Neonatology).
	Health facility assessment to examine the capacity of designated EmONC health facilities including the scope of emergency care currently being provided and existing staffing levels	Health facility managers (or representatives) and managers (or heads) of maternity or delivery wards	There are 112 designated EmONC health facilities. The health facility survey will require inputs from the health facility manager who will respond to general questions concerning the facility (e.g., infrastructure and staffing) and the manager of maternity or delivery wards who will answer specific questions concerning maternal and xnewborn care (e.g., EmONC signal functions). Therefore, the health facility survey will be conducted on 224 participants.
	Individual delivery care provider assessment to evaluate the extent to which they are confident to provide emergency care for maternal and newborn complications	Delivery care providers present in maternity or labour wards during our visits	There are an estimated 831 delivery care providers across all 112 designated EmONC health facilities (Supplement 2). Estimation of total delivery care staff in designated EmONC health facilities). We estimate to survey 40% of the possible sample (n = 335 participants) corresponding to delivery care providers who will be on duty during our visits.
Two	Secondary data analysis of historical records of graduates to examine the total national stock of delivery care professionals	—	—
	Secondary data analysis of historical records of recruits to explore the extent to which the government absorbs delivery care graduates	—	—
	Interviews with students in private medical universities and nursing and midwifery schools to estimate the financial cost of training different delivery care cadres using a payee-perspective (student) approach	Medical students and nursing and midwifery students in private universities and schools	There are 2 private medical schools in Burundi and both will be included in the study. Nursing and midwifery schools will be stratified in public versus private and urban versus rural to account for cost variability which depends on the geographic location of the school (e.g., private schools in major cities may charge higher costs to account for higher expenses such as investment in buildings). A random or convenient sample of 10 schools will be used in this study. Since tuition fee does not vary per student, we will survey a maximum of 5 random students in each selected university or school to ensure consistency in costs data (n=65 students).
	Secondary cost data analysis to estimate the financial cost (and increase in cost) of training and employing different delivery care cadres in public schools using a provider-perspective (government) approach	—	—
Three	A scoping review to understand what workforce gap analysis methods have been used in LMICs	—	—
	Analysis and costing of the workforce gap between existing and proposed * skilled delivery care staffing requirements for EmONC health facilities in Burundi	—	—
	Development of staffing trade-offs of different skill-mix options (e.g., nurses vs midwives; midwives vs junior doctors, etc.) over a realistic policy timeline (e.g., 10–15 years)	—	—
	A stakeholder panel to stimulate discussion and policy reform	Policymakers, donors, researchers, and implementers	6–8 participants (Supplement 1)

*Current delivery staff levels will be ascertained against proposed normative staffing standards agreed upon during the stakeholder discussion held earlier in phase one: *Stakeholder panel to discuss what EmONC staffing standards should be acceptable for Burundi*.

### Study description and methods

**Figure 1.**
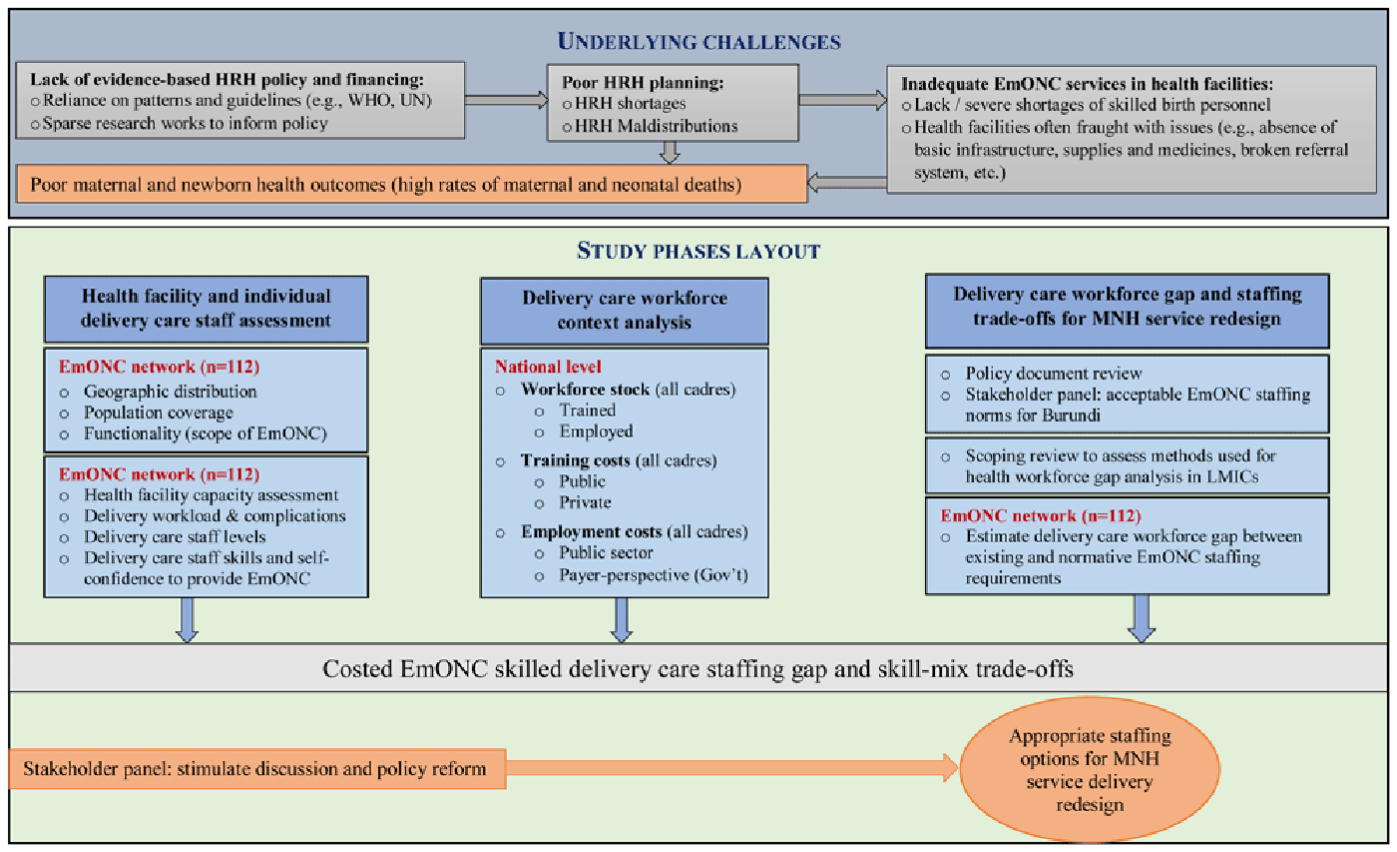
Overview of the MNH-Redesign study phases.

This study will be implemented in three phases as described in Figure 1.

## Underlying challenges

The study rationale stems from the challenges underpinning efficient HRH planning in Burundi. Like in most other low-income countries (LICs), Burundi lacks empirical local evidence to guide policy discussion, which implies that the country planning relies on the adoption of global guidelines or recommendations and on historical patterns which are incrementally revised ^28, 29^. For instance, the results of a study that mapped available health research works highlighted that Burundi suffers a severe lack of empirical evidence. From 2002 until 2011, only 34 health publications, which were mostly authored by foreign researchers, came from Burundi ^30^. In comparison, Rwanda contributed 205 health publications and Kenya a wealth of 3004 health publications ^30^. Most importantly, while Kenya and Rwanda respectively registered 205 and 20 clinical trials during the same period, Burundi did not conduct any clinical trial ^30^. Therefore, health workforce planning is poor and non-evidence-based, which undermines the country’s aspiration to deliver on the universal health coverage agenda ^18^.

### Delivery care staffing levels

Understanding current delivery care staffing levels in designated EmONC health facilities is critical as this will inform the workforce gap analysis to quantify real-time staffing needs. With reference to international standards for clinical staffing of delivery care in maternity units, the International Federation of Gynaecology and Obstetrics (FIGO) sets out minimum requirements for BEmONC and CEmONC birthing centres depending on the number of births. For instance, a BEmONC birthing centre with approximately 2000 deliveries should be equipped with at least three skilled birth attendants and a minimum of four labour beds and three individual delivery rooms. Ideally, such a health facility should be equipped with five skilled delivery attendants and seven individual labour and delivery rooms ^31^. We will map EmONC health facilities and conduct a health facility assessment to understand the scope of EmONC services being provided. The health facility assessment will enable appraisal of which EmONC facilities are functioning and providing which services with which skilled delivery staff levels. Next, we will conduct an individual delivery care provider survey to assess their general knowledge in maternal and newborn health and their self-confidence to provide emergency care for complications. Findings from the health facility and individual assessments will inform how well Burundi performs concerning EmONC services provision including skilled delivery personnel and further feed into the workforce gap analysis later in the study.

### Delivery care workforce context

Understanding Burundi’s capacity concerning the national stock of delivery care professionals constitutes another important input to inform policy change. In the first instance, we will assess the total stock of delivery care professionals by documenting historical numbers of graduates in both public and private medical and nursing schools. Next, we will document the training costs per type of cadre and per type of institution, in the public and private sectors. Direct financial training costs will be explored using a provider-perspective approach i.e., the government for public schools, and a payee-perspective approach i.e., trainees in private schools. Last, the same approach will be used to determine the government cost of employing each delivery care cadre. Employment costs will be stratified by cadre and by type of geographic residence to encompass rural incentives. The workforce stock analysis will help to understand the government budget impact to train and employ different delivery care cadres. This outcome will feed into the staffing gap costing exercise in the next study phase.

### Delivery care workforce gap and staffing tradeoffs

We will review policy documents to gather evidence on delivery care staffing standards or norms for EmONC health facilities, particularly in low-income settings. Results of the document review will be appraised against policy recommendations for EmONC staffing in Burundi. This stage will require the engagement of relevant stakeholders to discuss what should be “acceptable” staffing norms or standards for Burundi. Next, we will conduct a scoping review to build the body of evidence on the methods that have been used to estimate the gap of health workers, particularly in LMICs. Each method will be appraised to understand its strengths and weaknesses and how it can be applied to Burundi. The chapter will conclude with the analysis of the current staffing gap in all EmONC health facilities. Most studies used staff workload to estimates current and future staffing needs ^32– 34^. Other methods include the health care worker to population ratio, the service demand, the service-target, and the health and service needs approaches ^35, 36^. A gap analysis will be done and skill-mix staffing alternatives developed to close the identified gap ^37, 38^. Since we aim to generate evidence that can stimulate policy reform discussion, we will employ two different gap analysis methods that are likely to yield different results to give room to stakeholder trade-offs at times of decision making or policy discussion.

### Study outcomes

Results from the latter section will be used to develop delivery care staffing tradeoffs using a skill-mix approach. Staffing alternatives will be costed drawing on the costs of employment of different delivery care cadres from phase two and presented during a local stakeholder panel to discuss what should be the most appropriate staffing options considering the financial needs and country capacity. In addition to the thesis, study findings will be used by the MoH and its partners to strengthen EmONC health facilities with the provision of needed EmONC human resources, equipment, supplies and essential medicines as well as infrastructures well relevant. Findings will form the basis to advocate for actions needed to improve maternal and newborn health survival in Burundi. 

### Data description and analysis plan

Data will be sourced using a set of methodological approaches (Supplement 3) including i) secondary data collection, ii) primary health facility assessments, iii) primary surveys with students and delivery care providers, iv) direct observation, v) stakeholder discussion, and vi) evidence synthesis using structured and non-structured literature and policy document review. Data description and analysis plan are described in Table 2.

**Table 2.** Data description and analysis plan.

Data type	Data description and analysis plan
Health facility data	We will collect geocoordinates and use the existing 2021 database on population coverage which is available at the Ministry of Health in Burundi. These data will be used to layout the geographic distribution of EmONC health facilities and evaluate disparities in population coverage. The geographic analysis will be done in ArcGIS software. The scope of emergency obstetric and neonatal care will be assessed using a health facility assessment (Supplement 4). The literature provides a range of tools used to collect health facility-level data to assess service availability, readiness, and provision ^15, 39– 44^. Tools commonly assess general requirements for health facilities depending on the level of care, routine obstetric and newborn care, and basic and comprehensive EmONC, among others ^45^. Our tool was adapted from the EmONC Needs Assessments toolkit (Averting Maternal Death and Disability) ^39^, the World Health Organization (WHO) service availability and readiness assessment (SARA) ^43, 46^, and the Quality Evidence for Health System Transformation (QuEST) health facility assessment tool ^47^. The EmONC Needs Assessment toolkit, which was designed by the Averting Maternal Death and Disability (AMDD) program at Columbia University Mailman School of Public Health and adopted by WHO, UNFPA, and UNICEF ^39, 45, 48^ enables to classify each health facility in either of the three categories: i) comprehensive EmONC, ii) basic EmONC, or iii) not an EmONC health facility ^49, 50^. Data collection will require trained data collectors and field visits in all 112 designated EmONC health facilities in Burundi. Specifically, we will collect data on the availability of basic infrastructure, the availability and functionality of EmONC equipment, supplies and medicines, and overall and delivery care staff plus weekly pattern staff availability. Further, we will record the number of deliveries, complications, deaths, etc. from maternity registries. Health facility data will be described to define functioning EmONC health facilities and explore the overall staffing capacity to provide emergency care. Workload patterns will be depicted using a run chart.
Individual data in health facilities	A sample of 335 delivery care providers in maternity wards will be surveyed using Supplement 5. We will collect background information and perceptions on working conditions, quality of care, and self-confidence. Further, we will assess general clinical, maternal, and newborn care knowledge. We will conduct descriptive statistics and continue with analytical models to understand the level of confidence and knowledge and determine individual and health facility level factors that affect confidence and knowledge to provide emergency care. Individual questionnaire was developed using the EmONC skills training module and the EmONC practical guidelines. Tools were validated by the technical and stakeholder committees comprising of staff from the MoH, WHO, JICA, ABUNE, AGOB, UNICEF, UNFPA, and the University of Burundi.
National stock of delivery care cadres	We will map public and private medical and nursing and midwifery schools in Burundi and obtain historical records of the number of delivery care graduates over the recent past and the number of delivery care cadres employed by the public sector over the same period. In Burundi, the Ministry of Health is the certificate awarding body for nurses and midwives while the Ministry of Education certifies medical doctors and medical specialists. Therefore, the total number of graduates per annum will be obtained from those two ministries. Summary statistics will be done to estimate the prevailing national total stock and determine the government capacity to absorb graduate delivery cadres.
Training cost data	Training cost estimation will be done separately in public and private schools. Public medical and nursing/midwifery schools receive a full government subsidy and students are paid a monthly subsistence stipend while private schools run a profit-making model and do not receive government financial support. **Training cost in private schools:** individual students (n = 60) in private schools will be surveyed on direct training financial cost (Supplement 6. Section 2.B) which comprises inclusive tuition fees and living costs. Average training cost stratified per type of training will be calculated. **Training cost in public schools:** the financial costs of training account for recurrent and infrastructure costs ^51, 52^. We will not collect infrastructure costs; assuming that the government does not need to build additional medical and nursing training infrastructures in the short-run (we anticipate that results from the schools mapping will support this assumption). Recurrent costs include: i) teaching staff salaries, ii) non- teaching (administrative) staff salaries, and iii) student subsidies. The overarching question to answer is: what is the provider’s average financial cost of producing each type of delivery care cadre? Costs data at the University of Burundi will be obtained from the Department for Finance while costs data for public nursing and midwifery training will be obtained from the Ministry of Finance or Labour. We will collect the following types of data: i) gross teaching staff salaries (TSS) and non-teaching staff salaries (nTSS) per annum, ii) total number of students per year at school or faculty level, and iii) monthly student subsidies. We will collect time-series data to allow education costs growth estimation and time horizon projections (Supplement 6. Section 2.A). Concerning student subsidies, while nursing and midwifery trainees receive a standard package, medical students receive an increasing package depending on the level of training. From first to third-year medical training, students receive a government subsidy only while students in higher classes 4 ^th^–5 ^th^ year and 6th year receive an additional package to compensate for clinical duties. In the same perspective, residents are paid a higher subsidy. Therefore, costs will be disaggregated by type and level of medical education. The Average Cost (AC) of producing each of the delivery care cadres will be estimated using the following formula*: *AC _i_* = ( *TSS _yi_* + *nTSS _yi_* + *AS _i_*) * *n _i_* ; where: *AC _i_* = average cost of producing a delivery care cadre *i* *TSS _yi_* = average annual teaching staff salary for a student *i* *nTSS _yi_* = average annual non-teaching staff salary for a student *i* *AS _i_* = gross annual subsidy for student *i* *n _i_* = number of years of training for student *i*. *The formula does not account for repeating students.
Employment cost data	We will focus on the public sector as the aim is to estimate the government budget needs for employing different delivery care cadres (Supplement b. Section 2.C). We will assume that: • The government does not need to build new or expand health facilities or maternity wards • In-service performance-based incentives paid to providers by health facilities are not captured into the employment direct investment by the government • Employment costs calculation is based on a newly recruited and non-experienced staff (a recent graduate or career entry-level employee) Annual total financial costs of employing different delivery care cadres will be assessed using a payer- perspective i.e., the government. Inclusive costs for newly recruited non-experienced staff will be calculated by the type of cadre using gross annual wages, annual wage increase rate, pre-service and in-service training costs (e.g., EmONC training), etc. as follows: $EC_{j}=\sum_{i=1}^{n}X_{ji}\;;\;\text{where:}$ *EC _j_* = annual government total cost of employing a delivery care cadre *j* *X _ji_* = annual individual cost *i* [wages, pre-service and in-service training] for each delivery cadre *j*

## Discussion

Evidence-based health planning and resource allocation has been found to improve cost-effectiveness and reduce wastage within scarce and resource-constrained contexts ^53– 55^. Concerning the health workforce, many countries including LMICs have started to develop time horizon projections of health professionals to provide national policymakers with the evidence needed for HRH policy development ^56– 59^. As outlined earlier, Burundi lacks evidence on the real needs of skilled delivery care personnel to meet staffing standards and ensure that all labouring mothers including those in rural settings receive adequate and timely care including for emergency complications. Furthermore, the country suffers a dearth of knowledge on the existing national stock of health professionals and the capacity to produce and employ different delivery care cadres. This constitutes a barrier to appropriate planning, hence the reliance on the adoption of global guidelines or recommendations and historical patterns of health planning ^28, 29^. This study will be the first large-scale research in Burundi that will generate national-level evidence on the capacity of designated EmONC health facilities and the scope of care being provided. The study will diagnose human resources issues by estimating and costing the staffing gap of skilled delivery personnel and will further develop skill-mix staffing tradeoffs to close the identified gap. Moreover, the study will inform on the government investments needed for rolling out alternative skilled delivery staffing proposals. Findings from this study will be projected on a reasonable policy timeframe to enable a time horizon planning.

Potential strengths of this study are the early engagement of national stakeholders and the stakeholder panels which are planned to take place during the study implementation. Stakeholder engagement has become a cornerstone in health guidelines and policy development ^60– 62^. Amounting evidence supports that stakeholder buy-in bolsters policy acceptability and adoption into local practice ^63, 64^. We formed and engaged a local permanent stakeholder advisory committee early in the project design (Supplement 1). The committee is composed of policymakers from the Ministry of Health (MoH), donors represented by WHO and the Japan International Cooperation Agency (JICA), academicians and researchers from the University of Burundi, and national implementers represented by Association Burundaise pour le Bien-Etre Familial (ABUBEF). The first committee meeting was held during the study design to seek stakeholders’ inputs. The committee will continue to convene on regular basis which is a strategic approach to maintain engagement and stimulate a smooth policy discussion. In addition to the permanent committee, we plan to conduct two stakeholder panels where a wider stakeholder audience will be invited. The first panel will aim to discuss EmONC staffing standards that are feasible in Burundi, sourcing from the review of EmONC policy documents from LMICs and global and regional guidelines. The second panel will be held at the end of the study to discuss staffing alternatives and policy options. Moreover, the use of mixed methods constitutes a potential strength as evidence from mixed methods studies help to better apprehend complex problems and produce evidence in support of policy reform ^65^. In addition to its bolstering effect, early stakeholder engagement constitutes a strategy to mitigate potential implementation challenges such as access to secondary data and the conduct of primary health facility surveys.

Results from this study will be disseminated locally through stakeholder meetings and globally as publications in peer-reviewed journals and through presentations at relevant scientific meetings and conferences. Most importantly, findings from this study will be compiled in a thesis report to be submitted to the Nuffield Department of Medicine at Oxford University in fulfillment of the Doctor of Philosophy in Clinical Medicine by the primary and corresponding author.

## Ethics clearance

This study has been approved by the Human Research Ethics Committee of the Faculty of Medicine, University of Burundi (approval ref. FM/CE/01/M/2022) and the Oxford Tropical Research Ethics Committee (OxTREC approval reference: 516-22) (Supplements 7). All participants will sign a written informed consent form (Supplement 8). We will not collect participants’ identities. Plus, principles of confidentiality and anonymity will be guaranteed. Collected data will be kept on encrypted computers and backed up on WHO Burundi and MoH servers.

## Study status

At time of manuscript submission (June 22 ^nd^, 2022), the country stakeholder committee has started planning for fieldwork in target health facilities, but recruitment of study participants has not started. However, data collection in schools, universities, ministries, and individual students is underway.

## List of abbreviations

ABUBEF: Association Burundaise pour le Bien-Etre Familial

ABUNE: Burundian Association of Neonatology

AC: average cost

AGOB: Burundian Association of Gynaecology and Obstetrics

AMDD: averting maternal death and disability program at Columbia University Mailman School of Public Health

EmONC: emergency obstetric and neonatal care

FIGO: International Federation of Gynaecology and Obstetrics

GDP: Gross Domestic Product

HIS: Health Information System

HRH: human resource for health

JICA: Japan International Cooperation Agency

LICs: low-income countries

LMICs: low and middle-income countries

MoH: ministry of health

nTSS: non-teaching staff salaries

OBGYM: obstetrics and gynecology

OxTREC: Oxford Tropical Research Ethics Committee

QuEST: Quality Evidence for Health System Transformation

TSS: Teaching staff salaries

UNFPA: United Nations Population Fund

UNICEF: United Nations Children's Fund

WHO: World Health Organization

## Data Availability

Figshare. Supplements - Addressing the need for an appropriate skilled delivery care workforce in Burundi to support Maternal and Newborn Health Service Delivery Redesign (MNH-Redesign): a sequential study protocol. DOI: https://doi.org/10.6084/m9.figshare.20055257.v1 ^66^ This project contains the following underlying data: - Supplement 1. List of local stakeholders for the MNH-Redesign project - Supplement 2. Estimation of total delivery care staff in designated EmONC health facilities - Supplement 3. Data sources - Supplement 4. Health facility questionnaire - Supplement 5. Individual delivery care provider questionnaire - Supplement 6. Individual student and school questionnaire - Supplement 7. Written informed consent form - Supplement 8. Full study protocol Data are available under the terms of the Creative Commons Attribution 4.0 International license (CC-BY 4.0).
