# Supplementary material for: Addressing the need for an appropriate skilled delivery care workforce in Burundi to support Maternal and Newborn Health Service Delivery Redesign (MNH-Redesign): a sequential study protocol
Source: Wellcome Open Res. 2022 Sep 26;7:196. Originally published 2022 Jul 27. [Version 2] doi: 10.12688/wellcomeopenres.17937.2 (PMC9520631; doi:10.12688/wellcomeopenres.17937.2)
Supplement: Peer review history

# Peer review: Reviewer response for version 2

**DOI:** 10.21956/wellcomeopenres.20408.r52526

**Published:** 2022-09-30

**Authors:** Katherine Semrau

**Affiliations:** 1 Ariadne Labs, Brigham and Women's Hospital, Boston, MA, USA; 2 Harvard Medical School, Boston, MA, USA

Thank you for the review response and updated manuscript!

Is the study design appropriate for the research question?

Yes

Is the rationale for, and objectives of, the study clearly described?

Yes

Are sufficient details of the methods provided to allow replication by others?

Partly

Are the datasets clearly presented in a useable and accessible format?

Not applicable

Reviewer Expertise:

Maternal and newborn health; epidemiology; quality of care

I confirm that I have read this submission and believe that I have an appropriate level of expertise to confirm that it is of an acceptable scientific standard.

# Peer review: Reviewer response for version 2

**DOI:** 10.21956/wellcomeopenres.20408.r52528

**Published:** 2022-09-27

**Authors:** Ejemai Eboreime

**Affiliations:** 1 Department of Psychiatry, Faculty of Medicine and Dentistry, University of Alberta, Edmonton, AB, Canada

The authors have addressed my concerns

Is the study design appropriate for the research question?

Partly

Is the rationale for, and objectives of, the study clearly described?

Yes

Are sufficient details of the methods provided to allow replication by others?

Partly

Are the datasets clearly presented in a useable and accessible format?

Not applicable

Reviewer Expertise:

Implementation science, primary health care, mental health

I confirm that I have read this submission and believe that I have an appropriate level of expertise to confirm that it is of an acceptable scientific standard.

# Peer review: Reviewer response for version 1

**DOI:** 10.21956/wellcomeopenres.19875.r51935

**Published:** 2022-08-30

**Authors:** Merlin Willcox

**Affiliations:** 1 Primary Care, Population Sciences and Medical Education, University of Southampton, Southampton, UK

**Major comments:**

 This is an interesting study and protocol.

 There are two important issues which need to be considered by the authors.

**Firstly: the discussion states “the use of mixed methods constitutes a potential strength”**. However, the data description and analysis plan in Table 2 refers only to quantitative data and quantitative analyses. If qualitative data collection and analyses are planned, these should be added to Table 2 as a minimum. If not, the statement about mixed methods should be removed from the discussion.

 Examples of opportunities for qualitative research are as follows:

- Phase 1: health facility assessment: it is not clear whether these will be written questionnaires, an online survey, or qualitative interviews, or a mix. Please clarify. I would suggest that a subsample could be selected for qualitative interviews to ask about their experience of strategies to increase recruitment. It would be good to include a few district health officers in this too. You may find that several strategies have already been tried.
- Phase 2: Interviews with medical, midwifery and nursing students – it isn’t clear whether you will interview 5 in total in each school, or 5 of each category. I would recommend 5 of each category. Furthermore, will you only ask about tuition fees? Why not ask about accommodation and subsistence costs? And why not also conduct more in-depth interviews about their career intentions – whether they would want to work in the public sector (and what it would take for them to do so) or whether they are intending to work in the private sector or even abroad. It would be interesting to compare this with students in government training schools. Their career intentions may differ compared to those in private schools – eg if they are not saddled with a large debt, they may be able to afford to work in government health facilities for a lower salary. They may also be obliged to work in govt health facilities as a condition of their government scholarships?

**Secondly, the focus appears to be heavily on “staffing norms” and “staffing” which implies numbers of health workers and posts – rather than other potential solutions.** In the aims, the authors state: “Also, it takes a long time to develop the right workforce if an axpansion in service provision is needed”. No reference is given for this statement, and it may not be true, for the following reasons:

1. In many African countries, there is a paradox of unemployed doctors / nurses etc, because the number trained is greater than the number of posts available. This has clearly been documented in several countries e.g. see https://gh.bmj.com/content/7/Suppl_1/e008420. ^1^
  So the workforce could immediately be expanded in many countries by simply increasing recruitment and retention of existing health workers. The authors should acknowledge this and include methods to assess the number of unemployed health workers in Burundi. How many doctors / nurses / midwives are trained each year, and how many of these are able to find jobs in government health facilities? – I can see you should be able to address this in your assessment of “national stock of delivery care cadres”. It would be important to use qualitative methods to understand reasons for any differences observed between staff available and posts filled. E.g. are there vacant posts which have been advertised with no applicants? (and is this because of poor pay / working conditions, or because of a shortage of people with the right qualifications?) Or are there insufficient posts in health facilities?
2. The distribution of the existing workforce is often suboptimal, both geographically and between types of health facility. E.g. in Mali and Uganda, there are far more vacancies in primary care than in secondary care facilities – see https://human-resources-health.biomedcentral.com/articles/10.1186/s12960-015-0073-8. ^2^
  Redistributing the existing workforce could markedly improve service provision. I can see that the authors are planning to collect data on this in the “Health facility data” (Table 2). However, alongside existing staffing in health facilities, it would also be good to obtain a measure of “unmet need” for services in different areas. Eg how many mothers are delivering at home or in inappropriate facilities? This will not be captured by only measuring number of deliveries in each official facility.
3. Increasing pay / working conditions and also improving staff management (to reduce absenteeism) may be more effective, in certain conditions, than simply increasing numbers of staff. E.g. in many health centres I have visited in sub-Saharan Africa, there are very high rates of absenteeism, because pay is poor and people work as little as possible in their designated government health facility, in order to then go and earn more money in the private sector. Also, there are no sanctions for staff absenteeism as this is accepted to be the “norm”. Increasing the number of poorly-paid posts may do little or nothing to improve this situation. For example a health centre with 5 midwives (compared to 3 midwives) may still only have 1 midwife on duty at any one time, because the midwives may organise themselves to have a 1 in 5 rota (instead of a 1 in 3 rota). The methods should include ways of addressing this issue – eg qualitative interviews of staff and managers about how to reduce absenteeism and the potential cost of paying staff enough so that they do not feel the need to moonlight in the private sector.
4. Training “unskilled” staff to fill the gaps. In practice, in many countries, staff considered to be “unskilled” are managing patients in many health facilities (eg nursing assistants, midwifery assistants, traditional birth attendants). see https://human-resources-health.biomedcentral.com/articles/10.1186/s12960-015-0073-8 ^2^
  The methods should assess the extent of this practice in Burundi, and whether training them with essential skills could immediately improve management, while waiting for more midwives etc to be trained. Please clarify whether your health facility data will include these cadres.
  ​​​​
  There are also several typos and minor errors which need to be corrected (see below).

**Minor (specific) comments:**

 Abstract:

- Change “Throughout the study, we will engage stakeholders to provide input into what is reasonable staffing norms as well as feasible staffing alternatives within Burundi’s budget capacity” to “Throughout the study, we will engage stakeholders to provide input into what **are** reasonable staffing norms as well as feasible staffing alternatives within Burundi’s budget capacity”

Intro:

- Change “More specific local research also underscore the critical role of EmONC services.” to “More specific local research also **underscores** the critical role of EmONC services.”
- “With attention to the quantity of trained health personnel, the number of medical doctors per 10 000 population remains below the thresholdold (from 0.28 in 2010 to 1.00 in 2017)19.”
  Correct spelling of “threshold”.
  I think the authors are referring to the WHO threshold of 2.3 health workers per 1000 population (or 23 per 10,000). If so, add a reference to this. If not, add a reference for the “threshold” to which you are referring. Are you sure that the figures you quote are per 10,000 and not per 1,000? Also the WHO “threshold” is for all health workers not only for doctors. If you are going to compare figures for Burundi to the WHO “threshold” you need to include ALL trained health workers including nurses and midwives, etc.

Aims:

- Change “axpansion in service provision” to “ **expansion** in service provision”.
- Change “geograpgic and population coverage” to “ **geographic** and population coverage”.

Study design:

- Change “what is reasonable staffing norms” to “what **are** reasonable staffing norms”

Underlying challenges:

- Change “From 2002 until 2011, only 34 health publications; which were mostly authored by foreign researchers; came from Burundi” change to “From 2002 until 2011, only 34 health publications **,** which were mostly authored by foreign researchers **,** came from Burundi”

Top of p7:

- Most studies used staff workload to estimates current and future staffing needs” change to “Most studies used staff workload to **estimate** current and future staffing needs”

Table 1:

- Change “There is an estimated 831 delivery care providers” to “There **are** an estimated 831 delivery care providers”

Is the study design appropriate for the research question?

Partly

Is the rationale for, and objectives of, the study clearly described?

Yes

Are sufficient details of the methods provided to allow replication by others?

Partly

Are the datasets clearly presented in a useable and accessible format?

Not applicable

Reviewer Expertise:

Primary health care, global health, maternal and child health, human resources for health.

I confirm that I have read this submission and believe that I have an appropriate level of expertise to confirm that it is of an acceptable scientific standard, however I have significant reservations, as outlined above.

## References

[1] : Health workforce supply, needs and financial feasibility in Lesotho: a labour market analysis. *BMJ Glob Health* .7(Suppl 1) : 10.1136/bmjgh-2021-008420 10.1136/bmjgh-2021-008420 35609924PMC9131109

[2] : Human resources for primary health care in sub-Saharan Africa: progress or stagnation?. *Hum Resour Health* .2015;13: 10.1186/s12960-015-0073-8 76 10.1186/s12960-015-0073-8 26358250PMC4566492

# Response

**Published:** 2022-09-20

**Authors:** Desire Habonimana

**Affiliations:** University of Burundi, Burundi

Merlin Willcox

 APPROVED WITH RESERVATIONS

**Major comments:**

 This is an interesting study and protocol.

 There are two important issues which need to be considered by the authors.

**Firstly: the discussion states “the use of mixed methods constitutes a potential strength”**. However, the data description and analysis plan in Table 2 refers only to quantitative data and quantitative analyses. If qualitative data collection and analyses are planned, these should be added to Table 2 as a minimum. If not, the statement about mixed methods should be removed from the discussion.

Thanks so much for your feedback, a bold contrib ution. By mixed methods, we mean the mixture of strategies and approaches of sourcing information. For instance, we will use primary data (collected at health facility level and health care providers), secondary data ( collected from health facilities, ministries, and schools), stakeholder discussions (which will take the form of Focused-g roup discussion), and structured literature search (scoping review). These methods allowed us to used the term “mixed methods” hoping that we are scientifically correct.

 Examples of opportunities for qualitative research are as follows:

- Phase 1: health facility assessment: it is not clear whether these will be written questionnaires, an online survey, or qualitative interviews, or a mix. Please clarify. I would suggest that a subsample could be selected for qualitative interviews to ask about their experience of strategies to increase recruitment. It would be good to include a few district health officers in this too. You may find that several strategies have already been tried. Surveys will be quantitative and administered using direct interviews with health managers. The questionnaire will be software - based (KoboCollect) and will comprise of question s that aim to assess the health facility capacity using tools adapted from existing tools including The SARA tool and AMDD (see table 2, health facility level). The study does not intent to interview health facility/ district health officers concerning strategies to increase human resources as policies to increase human resources are taken at higher decision-making levels. In other words, in Burundi, the central level of the Ministry of Health is the leading body to handle human resources strategies. Lower cadres (at peripheral levels) do not take part to decision making. That is why our aim is to organise stakeholder discussions rather than interviewing lower cadres.
- Phase 2: Interviews with medical, midwifery and nursing students – it isn’t clear whether you will interview 5 in total in each school, or 5 of each category. I would recommend 5 of each category. Furthermore, will you only ask about tuition fees? Why not ask about accommodation and subsistence costs? And why not also conduct more in-depth interviews about their career intentions – whether they would want to work in the public sector (and what it would take for them to do so) or whether they are intending to work in the private sector or even abroad. It would be interesting to compare this with students in government training schools. Their career intentions may differ compared to those in private schools – eg if they are not saddled with a large debt, they may be able to afford to work in government health facilities for a lower salary. They may also be obliged to work in govt health facilities as a condition of their government scholarships? Thanks f or this invaluable contribution. We will interview 5 students in each school which exactly corresponds to more than 5 students in each c ategory of students. In fact, we will interview 5 medical students in each of the two private Universities, 5 students in each of the remaining 10 schools (some schools train only midwives or nurses while others train both). In private schools, we will collect data on inclusive tuition fees as well as living costs (accommodation, meals, other bills). Our study will unfortunately assess career intentions as this slightly lies beyond our scope.

**Secondly, the focus appears to be heavily on “staffing norms” and “staffing” which implies numbers of health workers and posts – rather than other potential solutions.** In the aims, the authors state: “Also, it takes a long time to develop the right workforce if an axpansion expansion in service provision is needed”. No reference is given for this statement, and it may not be true, for the following reasons: We totally agree with you on this. In many low-income countries, the number o f graduates largely outweighs the number of employed, mostly due to incapacity of the government / private sector to absorb graduates. By alluding to “ it takes a long time to develop the right workforce if an expansion in service provision is needed”, we mean that it takes long to train health care professionals. We have changed the word “develop” and made it more specific “ produce ”.

1. In many African countries, there is a paradox of unemployed doctors / nurses etc, because the number trained is greater than the number of posts available. This has clearly been documented in several countries e.g. see https://gh.bmj.com/content/7/Suppl_1/e008420. ^1^
  So the workforce could immediately be expanded in many countries by simply increasing recruitment and retention of existing health workers. The authors should acknowledge this and include methods to assess the number of unemployed health workers in Burundi. How many doctors / nurses / midwives are trained each year, and how many of these are able to find jobs in government health facilities? – I can see you should be able to address this in your assessment of “national stock of delivery care cadres”. It would be important to use qualitative methods to understand reasons for any differences observed between staff available and posts filled. E.g. are there vacant posts which have been advertised with no applicants? (and is this because of poor pay / working conditions, or because of a shortage of people with the right qualifications?) Or are there insufficient posts in health facilities? Certainly, we will evaluate the national stock which is the difference between available and employed delivery care professionals. Evaluation of this stock will be done using historical records (over a period of 10 or so years depending on available data) of the number of delivery care professionals who graduated each year from all the universities and schools minus the number of these professionals who have been recruited in each corresponding year. We have acknowledged that we will not account for those entering private sector (NGOs, private health facilities, industry, brain drain, etc.) due to inability of finding an accurate method to do so. Plus, except those joining loc al private health facilities, the other professionals less profit delivery care as they either work in NGOs (without direct impact on delivery care), industries (e. g., insurance companies), or choose to leave the country.
2. The distribution of the existing workforce is often suboptimal, both geographically and between types of health facility. E.g. in Mali and Uganda, there are far more vacancies in primary care than in secondary care facilities – see https://human-resources-health.biomedcentral.com/articles/10.1186/s12960-015-0073-8. ^2^

  Redistributing the existing workforce could markedly improve service provision. I can see that the authors are planning to collect data on this in the “Health facility data” (Table 2). However, alongside existing staffing in health facilities, it would also be good to obtain a measure of “unmet need” for services in different areas. Eg how many mothers are delivering at home or in inappropriate facilities? This will not be captured by only measuring number of deliveries in each official facility. We will measure unmet needs by i) comparing available staff versus “ minimum standard s ” (there are minimum standards for EmONC health facilities e.g., FIGO standards https://obgyn.onlinelibrary.wiley.com/doi/abs/10.1002/ijgo.12815 a nd Burundi has also a policy that establishes minimum standards for EmONC health facilities) and versus “ workload” using the Workload Indicators of Staffing Need (WISN) which is seen as an accurate metrics to understand the “unmet staffing needs”. Plus, we might find it needed / useful to use the existing “expected deliveries” to triangulate information and estimate the “ unmet needs”. However, existing data on estimated deliveries are disaggregated by health district and not health facility level, making it less informative in our stance.
3. Increasing pay / working conditions and also improving staff management (to reduce absenteeism) may be more effective, in certain conditions, than simply increasing numbers of staff. E.g. in many health centres I have visited in sub-Saharan Africa, there are very high rates of absenteeism, because pay is poor and people work as little as possible in their designated government health facility, in order to then go and earn more money in the private sector. Also, there are no sanctions for staff absenteeism as this is accepted to be the “norm”. Increasing the number of poorly-paid posts may do little or nothing to improve this situation. For example a health centre with 5 midwives (compared to 3 midwives) may still only have 1 midwife on duty at any one time, because the midwives may organise themselves to have a 1 in 5 rota (instead of a 1 in 3 rota). The methods should include ways of addressing this issue – eg qualitative interviews of staff and managers about how to reduce absenteeism and the potential cost of paying staff enough so that they do not feel the need to moonlight in the private sector. We perfectly agree these observations and suggestions. In many contexts, absenteeism rate is high due to moonlighting. That is the reason why we have proposed to collect pattern data on weekly availability (weekdays days versus nights, weekends / holidays days versus nights) of each staff cat egory that is available in each health facility. However, strat egies to address shortages of staff (bother the numbers and availability on duty) will be discussed during stakehol der meetings. Our overall aim is to engage policy discussions (by stakeholders) to ensure appropriateness of the project outcomes going forward.
4. Training “unskilled” staff to fill the gaps. In practice, in many countries, staff considered to be “unskilled” are managing patients in many health facilities (eg nursing assistants, midwifery assistants, traditional birth attendants). see https://human-resources-health.biomedcentral.com/articles/10.1186/s12960-015-0073-8 ^2^
  The methods should assess the extent of this practice in Burundi, and whether training them with essential skills could immediately improve management, while waiting for more midwives etc to be trained. Please clarify whether your health facility data will include these cadres. Exactly, this is absolutely true. Our study will collect information on training received (preboarding or in-service training) on each delivery care in all health facilities. This information will be supplemented by an indicator questionnai re of a selected sample of delivery care professionals to assess their knowledge of “complications management” as well as their self-confidence to manage maternal and / or neonatal complications. The individual questionnaire was designed using the locally adapted EmONC guidelines and training module which clearly set out key skills delivery care personnel should have.
  ​​​​
  There are also several typos and minor errors which need to be corrected (see below).

**Minor (specific) comments:**

 Abstract:

- Change “Throughout the study, we will engage stakeholders to provide input into what is reasonable staffing norms as well as feasible staffing alternatives within Burundi’s budget capacity” to “Throughout the study, we will engage stakeholders to provide input into what **are** reasonable staffing norms as well as feasible staffing alternatives within Burundi’s budget capacity” Thank you. We have made the change.

Intro:

- Change “More specific local research also underscore the critical role of EmONC services.” to “More specific local research also **underscores** the critical role of EmONC services.” Thank you. We have made the change.
- “With attention to the quantity of trained health personnel, the number of medical doctors per 10 000 population remains below the thresholdold (from 0.28 in 2010 to 1.00 in 2017)19.” Thank you. We have made the change.
  Correct spelling of “threshold”.
  I think the authors are referring to the WHO threshold of 2.3 health workers per 1000 population (or 23 per 10,000). If so, add a reference to this. If not, add a reference for the “threshold” to which you are referring. Are you sure that the figures you quote are per 10,000 and not per 1,000? Also the WHO “threshold” is for all health workers not only for doctors. If you are going to compare figures for Burundi to the WHO “threshold” you need to include ALL trained health workers including nurses and midwives, etc. Thank you. We have made the change.

Aims:

- Change “axpansion in service provision” to “ **expansion** in service provision”. Thank you. We have made the change.
- Change “geograpgic and population coverage” to “ **geographic** and population coverage”. Thank you. We have made the change.

Study design:

- Change “what is reasonable staffing norms” to “what **are** reasonable staffing norms” Thank you. We have made the change.

Underlying challenges:

- Change “From 2002 until 2011, only 34 health publications; which were mostly authored by foreign researchers; came from Burundi” change to “From 2002 until 2011, only 34 health publications **,** which were mostly authored by foreign researchers **,** came from Burundi” Thank you. We have made the change.

Top of p7:

- Most studies used staff workload to estimates current and future staffing needs” change to “Most studies used staff workload to **estimate** current and future staffing needs” Thank you. We have made the change.

# Peer review: Reviewer response for version 1

**DOI:** 10.21956/wellcomeopenres.19875.r51936

**Published:** 2022-08-19

**Authors:** Katherine Semrau

**Affiliations:** 1 Ariadne Labs, Brigham and Women's Hospital, Boston, MA, USA; 2 Harvard Medical School, Boston, MA, USA

Thank you for the chance to review. The authors have presented an important justification and a set of research questions to address the needs and opportunities for skilled birth attendance provision in Burundi. The sequential study will use a three-phase approach with a (i) document/policy review; (ii) cross sectional study on health facilities and staffing; and (iii) costing study. There are a few areas where clarification would be useful to readers:

1. The justification for the study is well-presented and strong. It would be helpful if the authors clarified that the facilities and training schools included are a census (i.e. all) or a sample? If they are a sample, how are they being selected? For example, in Table 1, the authors share that there are 2 private medical schools and allude to several nursing/midwifery schools; how were they selected? Are there government/public schools as well?
2. In the Introduction, the authors presented the number of medical doctors per 1000 population that remains below the threshold, but the readers may not know what that recommended level/threshold is. Please consider adding the background. 
3. In the Methods section, the authors highlight a stakeholder group and then add more detail in the Discussion. Please add the details in the Methods as it will make it clearer who is included in the stakeholder group and how they were selected.
4. The delivery care staffing levels information will be critical to the study and recommendations. The authors need to provide more details about how the surveys will be developed and topics areas to understand the skills that will be covered. Are there validated tools being used? Additional detail about tool development would be useful. Further, it is known that provider confidence and knowledge alone are necessary, but insufficient, to provide high quality care. Did the authors consider assessment of infrastructure and supply availability that may prevent high quality care provision? 
5. Please make sure to run a spellcheck throughout the manuscript and also check for references to the dissertation/thesis. In addition to the thesis, I understand there are other uses for these data. It would be important to note who will receive and use this information in the Study outcomes section.
6. In Table 2, the authors note the assumption that the government does not need to build new or expand health facilities. Will the authors re-consider their assumptions after Phases I and II before embarking on Phase III?

Is the study design appropriate for the research question?

Yes

Is the rationale for, and objectives of, the study clearly described?

Yes

Are sufficient details of the methods provided to allow replication by others?

Partly

Are the datasets clearly presented in a useable and accessible format?

Not applicable

Reviewer Expertise:

Maternal and newborn health; epidemiology; quality of care

I confirm that I have read this submission and believe that I have an appropriate level of expertise to confirm that it is of an acceptable scientific standard, however I have significant reservations, as outlined above.

# Response

**Published:** 2022-09-20

**Authors:** Desire Habonimana

**Affiliations:** University of Burundi, Burundi

Katherine Semrau

 APPROVED WITH RESERVATIONS

 info_outline

 Thank you for the chance to review. The authors have presented an important justification and a set of research questions to address the needs and opportunities for skilled birth attendance provision in Burundi. The sequential study will use a three-phase approach with a (i) document/policy review; (ii) cross sectional study on health facilities and staffing; and (iii) costing study. There are a few areas where clarification would be useful to readers:

1. The justification for the study is well-presented and strong. It would be helpful if the authors clarified that the facilities and training schools included are a census (i.e. all) or a sample? If they are a sample, how are they being selected? For example, in Table 1, the authors share that there are 2 private medical schools and allude to several nursing/midwifery schools; how were they selected? Are there government/public schools as well?

Thanks for this note. In Burundi, the EmONC network is made of 112 health facilities of which 59 BEmONC and 53 CEmONC. All these 112 facilities will be included in ou r study. Concerning ministries, data will be collected from three relevant ministries hold ing data on education of delivery care professionals (doctors, midwives, and nurses) including cost of training: ministry of health, ministry of education, and ministry of finance. There are one public University (University of Burundi) and two private universities (University of Ngozi and Hope Africa University) training medical doctors, and all will be included in the study to understand the cost of training as well as estimate the national stock (trained doctors over time ). T here are about 18 public and private schools training nurses and midwives. To appraise the cost of training, we will stratify these schools into public vs private and again into rural vs urban as costs vary according to these aspects. A subset of rural public, rural private, urban public and urban priva te schools will be selected (convenient or random if there are more than one school in each stratum ) and used as a sample for the study. We anticipate surveying a sample of 10 schools in total.

1. In the Introduction, the authors presented the number of medical doctors per 1000 population that remains below the threshold, but the readers may not know what that recommended level/threshold is. Please consider adding the background. 

There are mainly three different thresholds used to assess health care workforce availability. They are:

1. The minimum of 2.3. skilled health workers (physicians and nurses/ midwives) per 1000 population, which was considered generally necessary to attain high coverage (80%) of skilled birth attendance ( The 2006 World health report )

2. The minimum workforce availability of 3.4 skilled health workers per 1000 population, termed as a “staff access deficit indicator”, which value has subsequently been updated to 4.1 per 1000 ( ILO definition)

3. Th e minimum of 5.9 skilled health professionals (midwives, nurses and physicians) per 1000 population which was identified as the workforce requirement for the Ending Preventable Maternal Deaths initiative (ENAP and EPMM) . 

W e used the threshold of 2.3 skilled health workers per 1000 population.

1. In the Methods section, the authors highlight a stakeholder group and then add more detail in the Discussion. Please add the details in the Methods as it will make it clearer who is included in the stakeholder group and how they were selected.

Thanks for asking to update the methods section with additional information on the stakeholder group. We have added details in Table 1 and updated the list of abbrevia tions.

1. The delivery care staffing levels information will be critical to the study and recommendations. The authors need to provide more details about how the surveys will be developed and topics areas to understand the skills that will be covered. Are there validated tools being used? Additional detail about tool development would be useful. Further, it is known that provider confidence and knowledge alone are necessary, but insufficient, to provide high quality care. Did the authors consider assessment of infrastructure and supply availability that may prevent high quality care provision? 

The health facility survey ( supplement 4) details information that will be collected. We will collect a wide range of data including infrastructure , equipment , supplies a nd drugs, etc, etc. that define a health facility and further a maternity that delivers care for complications. Surveys development was informed by existing tools ( Table 2. Health facility data description provides a note of tools that inspired development of health facility survey tools ) and adapted to Burundi’s context. We have updated the ‘individual questionnaire’ section to include details on its development and validation (Table 2. Individual data in health facilities description). 

1. Please make sure to run a spellcheck throughout the manuscript and also check for references to the dissertation/thesis. In addition to the thesis, I understand there are other uses for these data. It would be important to note who will receive and use this information in the Study outcomes section.

Thanks for the suggestion to run spellcheck. We have checked spelling throughout the manuscript. Plus, we have updated the study outcome section to mention wh at else the study findings will be used for in addition to the thesis .

1. In Table 2, the authors note the assumption that the government does not need to build new or expand health facilities. Will the authors re-consider their assumptions after Phases I and II before embarking on Phase III?

Though this assumption was made, stakeholder discussion (based on survey findings) may suggest expansion of infrastructures where relevant. However, infrastructure expansion is something hard to achieve in the short run, hence our assumptions. Markedly, o ur study aims to propose immediate and easy-to-implement actions.

# Peer review: Reviewer response for version 1

**DOI:** 10.21956/wellcomeopenres.19875.r51938

**Published:** 2022-08-12

**Authors:** Ejemai Eboreime

**Affiliations:** 1 Department of Psychiatry, Faculty of Medicine and Dentistry, University of Alberta, Edmonton, AB, Canada

This is a very interesting study proposed.

 My key comment is with respect to how the proposed methods are described. I think more detail and/or better organization of the detail would be helpful. For example, the authors describe that the study will be implemented in three sequential phases:

1. an initial policy document review to explore global norms and local policy intentions for BEmONC and CEmONC in Burundi with a particular focus on stated or reasonable staffing norms for different levels of the facility 
2. a cross-sectional survey of all BEmONC and CEmONC health facilities to map out and determine what percent of facilities are functional including geographic and population coverage gaps, to identify staffing gaps assessed against norms or expectations, and to identify other needs for health facility strengthening. 
3. examine potential staffing costs and explore alternative staffing options (service designs) that might best promote service delivery with adequate budget impacts to increase efficiency.

I think that it would be a very good idea to organize the description of the methodological detail along these subheadings. In the current form, the details are not sequential, making it a little challenging for readers to follow

Is the study design appropriate for the research question?

Partly

Is the rationale for, and objectives of, the study clearly described?

Yes

Are sufficient details of the methods provided to allow replication by others?

Partly

Are the datasets clearly presented in a useable and accessible format?

Not applicable

Reviewer Expertise:

Implementation science, primary health care, mental health

I confirm that I have read this submission and believe that I have an appropriate level of expertise to confirm that it is of an acceptable scientific standard, however I have significant reservations, as outlined above.

# Response

**Published:** 2022-09-20

**Authors:** Desire Habonimana

**Affiliations:** University of Burundi, Burundi

Ejemai Eboreime

 APPROVED WITH RESERVATIONS

 info_outline

 This is a very interesting study proposed.

 My key comment is with respect to how the proposed methods are described. I think more detail and/or better organization of the detail would be helpful. For example, the authors describe that the study will be implemented in three sequential phases:

1. an initial policy document review to explore global norms and local policy intentions for BEmONC and CEmONC in Burundi with a particular focus on stated or reasonable staffing norms for different levels of the facility 
2. a cross-sectional survey of all BEmONC and CEmONC health facilities to map out and determine what percent of facilities are functional including geographic and population coverage gaps, to identify staffing gaps assessed against norms or expectations, and to identify other needs for health facility strengthening. 
3. examine potential staffing costs and explore alternative staffing options (service designs) that might best promote service delivery with adequate budget impacts to increase efficiency.

I think that it would be a very good idea to organize the description of the methodological detail along these subheadings. In the current form, the details are not sequential, making it a little challenging for readers to follow

**Thank yo u so much for this contribution which we gratefully accept. We would like to highlight that these phases do not follow the chronological order though information from each phase inputs into the other. This means that we can start with cross-sectional surveys and continue with the scoping review and vi c e-versa; for in stance. Therefore, our methods section sought to highlight the following areas: **

1.
  1. **Study design**
  2. **Study settings and participants **
  3. **Descrip tion of the study methods. This is where we describe the framework which comprises the three study phases: i) cross-sectional surveys conducted in health facilities and on delivery care personnel , ii) delivery care workforce context analysis , and iii) delivery care workforce gap analysis. These sections are succinct descriptions of each phase by laying out the methods to be used and the expected outputs. Overall, outputs from all phases will merge together to enable the costing of delivery care unmet needs which will feed into policy discussions. **
  4. **Study outcomes **
  5. **Data description and analysis plan**

**We believe that point 3 (description of the study methods) can be seen as structured to respond to the layout as per the study phases. However, we** **are keen on your feedback, and we accept any further comment with this regard. Thank you.**
